# Association between midlife health behaviours and transitions out of employment from midlife to early old age: Whitehall II cohort study

**DOI:** 10.1186/s12889-016-3970-4

**Published:** 2017-01-17

**Authors:** Gareth Hagger-Johnson, Ewan Carr, Emily Murray, Stephen Stansfeld, Nicola Shelton, Mai Stafford, Jenny Head

**Affiliations:** 1 Administrative Data Research Centre for England (ADRC-E), University College London, London, WC1E 6BT UK; 2 Department of Epidemiology and Public Health, University College London, London, UK; 3 Wolfson Institute of Preventive Medicine, Queen Mary University of London, London, UK; 4 MRC Unit for Lifelong Health & Ageing, University College London, London, UK

**Keywords:** Alcohol drinking, Cigarette smoking, Employment, Health behaviour, Retirement

## Abstract

**Background:**

It is important to determine whether unhealthy behaviours might influence transitions out of employment from midlife to old age, given the anticipated need for adults to work for longer. Our aim was to determine the association between repeated assessments of cigarette smoking, heavy/problem alcohol drinking, low physical activity and poor diet at midlife, in relation to work exit from midlife to old age.

**Methods:**

Data from 7704 participants (5392 men) from the Whitehall II cohort study in employment at midlife were used to evaluate the association between unhealthy behaviours and a subsequent transition out of work during 22 years follow-up, using logistic regression models.

**Results:**

Men who smoked cigarettes, consistently drank alcohol heavily, or reported problem drinking, were more likely to leave employment over follow-up. Women with a consistently poor diet were more likely to leave employment. Associations were stronger when the reason for leaving was health grounds, and stronger among those with persistently unhealthy behaviours over follow-up. The size of the effects were broadly equivalent to one advancing year of age on employment. Physical health functioning over follow-up only partly accounted for the associations with work exit, whereas physical and mental functioning accounted for most of the associations with work exit on health grounds.

**Conclusions:**

Unhealthy behaviours in midlife are associated with transitions out of employment into old age. Promoting healthy behaviours at midlife might support current policy initiatives aimed at extending working life. Future research should consider possible mechanisms that link behaviours to transitions out of employment, and consider sex differences in larger cohorts.

**Electronic supplementary material:**

The online version of this article (doi:10.1186/s12889-016-3970-4) contains supplementary material, which is available to authorized users.

## Background

There is increasing scientific and policy interest in discovering determinants of early exit from the labour market [1]. Although many variables are not under individual control (e.g. taxation, immigration, recession, government policy), health behaviours such as cigarette smoking, heavy or problematic alcohol drinking, low levels of physical activity and poor diet, may result in lower earnings [2], withdrawal from the labour market, or early retirement on health grounds. Since health behaviours are modifiable, it is important to understand how they influence work and withdrawal from work in later life, both for empirical and public health policy reasons [3]. It is known that unhealthy behaviours in adults of working age create risks for employees’ own later health [4] (e.g. retirement on health grounds, premature mortality before retirement), can worsen socio-economic inequalities [5], and have costs for employers (e.g. sickness absence pay, loss of skills and experience following early retirement) [6, 7]. Less is known about whether unhealthy behaviours contribute to subsequent transitions out of work considered from midlife through to old age, which would be important to know when considering policies designed to encourage extended working.

Previous studies have found that smoking [1, 8–16] and low physical activity [10, 11, 15–18] are associated with transitions out of work on health grounds. There is emerging evidence that heavy alcohol drinkers [2, 10, 19–21], problem drinkers [2, 19, 22] and non-drinkers [20, 22, 23] are more likely to retire on health grounds compared to moderate drinkers; the latter group thought to include former heavy drinkers who may have stopped drinking for health reasons. Evidence from twin studies suggests that these effects are independent from shared environmental and genetic determinants of health behaviour [1, 2]. Associations between obesity [24, 25] and transitions out of employment suggest that modifiable risk factors earlier in adulthood might influence future work exit through changes in physical health. There is some evidence that patterns may differ for men and women. For example in one study, the association between physical activity and disability retirement was stronger in men than in women [17]. No study has considered whether repeated assessments of all four health behaviours across midlife are associated with transitions out of employment from midlife to old age.

The aim of our study was to determine the association between accumulated unhealthy behaviours (cigarette smoking, heavy alcohol use, physical inactivity, and poor diet) at midlife, in relation to transitions out of work from midlife to old age, in a large occupational cohort who had the option to retire and continue working beyond pensionable age. Additionally, we sought to determine whether the effects were different for men and women.

## Method

### Design

Data are drawn from the Whitehall II prospective cohort study [26]. At recruitment in 1985/88 (age 35 to 55), participants were all employed across 20 civil service departments in London. All participants provided written consent and the University College London ethics committee approved this study. Normal retirement age for the cohort was 60, but early retirement options were available and participants could obtain employment elsewhere. Data on health behaviours (cigarette smoking, alcohol consumption, physical activity level and daily fruit/vegetable consumption) were recorded three times across the proposed exposure window (age range 35 to 64) in 1984/85, 1989/90, 1991/93. Details can be found in the cohort profile [26] and in the questionnaires themselves (available from http://www.ucl.ac.uk/whitehallII/data-sharing). To reduce bias, missing data on health behaviours were imputed with values from adjacent observations, where available. For the current study, participants were eligible for inclusion if they remained in full time employment in 1991/93 (study baseline). Employment status was recorded in questionnaires during seven follow-ups (age range 45 to 85) in 1995/96, 1997/99, 2001, 2002/04, 2006, 2007/09 and 2012/13, allowing us to determine transitions out of employment. Health behaviours were recorded another four times (1997/99, 2002/04, 2007/09, 2012/13) allowing us to consider the influence of health behaviour change over follow-up.

### Measures

#### Transition out of work

Over follow-up, participants were considered to be in employment if they were still working in the civil service, or were in paid employment elsewhere (full or part time). Participants who were no longer working at a subsequent follow-up were classified as having transitioned out of work.

#### Health behaviours

Physical activity was defined as high (>2.5 h/week of moderate or >1 h/week of vigorous physical activity, low (<1 h/week moderate and <1 h/week vigorous physical activity) or medium if falling between these two categories. Alcohol consumption was grouped into non-drinker, moderate drinker (1-14/1-21 alcohol units/week for women/men, or heavy drinker (>14/>21 units/week) [27]. Problem drinking was assessed using the cutting down, annoyance by criticism, guilty feeling, and ‘eye-opener’ (CAGE) questionnaire (1991/93 only) [28]. This has four previously validated questions recording alcohol drinking behaviour (response options ‘yes’ or ‘no’). Participants were classified as problem drinkers if they answered ‘yes’ to two or more questions. Poor diet was defined as fruit/vegetable consumption less than daily (vs. at least daily). Smoking status was classified as current/intermittent smoker, ex-smoker or non-smoker.

To evaluate cumulative exposure to smoking, participants were classified into never smokers, ex-smokers at recruitment (1984/95), and current or intermittent smokers. For alcohol drinking, physical activity and poor diet, participants were grouped according to how many of three midlife observations (1984/85, 1989/90, 1991/93) they reported heavy alcohol drinking, no alcohol drinking, low physical activity or poor diet (separately for each health behaviour).

#### Covariates

Covariates were age, sex, socio-economic status, and chronic disease at baseline. Socio-economic status was obtained from employment grade, considered a comprehensive marker of level of responsibility, status and income [26]. Chronic disease was defined as prevalent coronary heart disease, stroke, cancer or diabetes, based on self-reported doctor diagnosed conditions. The Short Form Health Survey-36 (SF-36) was additionally available for most participants over follow-up, which we used for supplementary analyses. It comprises eight scales: physical functioning, role limitations attributable to physical problems, social functioning, bodily pain, general mental health, role limitations attributable to emotional problems, vitality, and general health perceptions. The scores are summarised into two components which have been previously validated: Physical Component Summary (PCS) and Mental Component Summary (MCS) [29]. For supplementary analyses evaluating the possible role of emotional and material adversity in childhood, a score was calculated (range 0, 10) from ten items: ‘you spent four or more weeks in hospital’, ‘your parents were divorced’, ‘your father/mother were unemployed, when they wanted to be working’, ‘your parent(s) were mentally ill or drank so often that it caused family problems’, ‘you were physically abused by someone close to you’, ‘your parents very often argued or fought’ and ‘you were in an orphanage/children’s home’. For supplementary analyses evaluating the role of cognitive ability (intelligence), Mill Hill vocabulary score [30] was available from validated cognitive tests administered in supervised conditions, three times (1997/99, 2002/04, 2007/09). The average score was treated as a measure of cognitive ability. We also considered the possible role of ethnic group as a covariate and effect modifier, which was classified as ‘ethnic minority’ or ‘White’.

### Statistical analysis

Descriptive statistics were used to evaluate differences in study variables between those experiencing a transition out of work compared to those remaining in work. For the main analysis, we used a discrete-time event history model [31]. This models the conditional probability (converted to odds ratios) of work exit in each discrete time period between study observations, broadly equivalent to the hazard rate of work exit, using a multilevel structure. This acknowledges that there are repeated measures of the same individual over time, allows missing data, and allows individually varying times of observation. Models were run in Stata version 14.0 as logistic regressions, with a robust cluster variance estimator to obtain correct standard errors. All four health behaviours were entered into the same model, minimally adjusting for age, then additionally adjusting for remaining covariates. In a separate model, we also considered problem alcohol drinking (in 1991/93) in a subsample of participants with available data on CAGE in 1991/93, adjusting for the covariates and other three health behaviours. Tests of global interaction (likelihood ratio test) were first used to evaluate whether the association between each health behaviour (considered separately) and a transition out of work differed for men/women. In sensitivity analyses, we repeated the analysis after excluding participants who transitioned out of work more than once, to evaluate if these employment patterns had biased the results. We also repeated the analysis using only transitions out of full time work, rather than full or part time work. We also reran the analyses after excluding participants who changed their health behaviours over follow-up (defined as movement from an unhealthy to a healthy behaviour) to consider the possible impact of health behaviour change over follow-up on employment patterns. We then evaluated the extent to which associations between unhealthy behaviour and transitions out of work might be explained by changes in health status over follow-up, by evaluation the percentage attenuation in the associations after the SF-36 physical and mental health component summary scores were added to the models. In supplementary analyses on subgroups of the analytic sample with available data, we considered the role of childhood adversity and cognitive ability by evaluating the extent to which associations between health behaviours and exit from employment were attenuated when added to the models. We also additionally adjusted the models for ethnic minority status, and evaluated whether associations were different for ethnic minorities.

## Results

The analytic sample comprised 7704 participants (28,976 person-time observations) with available data on behaviours at midlife, employment status over follow-up, and covariates. Compared to those in the study population at recruitment excluded from the analytic sample due to missing data or attrition, the analytic sample comprised fewer females (30.0% vs. 42.3%, *p* < 0.001), slightly younger participants (44.7% vs. 45.7%, *p* < 0.001), fewer participants with low socio-economic status (18.1% vs 36.3%, *p* < 0.001), fewer current smokers (16.9% vs. 22.6%, *p* < 0.001), but a slightly higher proportion of heavy alcohol drinkers (16.4% vs. 14.5%, *p* = 0.02) and a higher proportion with poor diet (59.5% vs. 53.1%, *p* < 0.001). Preliminary analysis showed that effects were significantly different for men and women (all *p* < = 0.03), leading us to separate men and women for analysis. Of the analytic sample, 6282 experienced a transition out of employment. There were 502 (6.5%) participants who returned to employment then transitioned out again, and 32 (0.4%) who experienced three transitions.

As shown in Table 1, participants who transitioned out of work tended to older, comprised more women, and those with chronic disease. Heavy alcohol drinkers were more likely to transition out of work, but cigarette smokers, those with low physical activity and poor diets were less likely to leave work. These patterns should be interpreted with caution because they have not been adjusted for likely confounding factors, particularly socio-economic status.

**Table 1:** Descriptive statistics for study variables at baseline

	Remained in full-time employment (*n* = 1422)	Transition out of work (*n* = 6282)	*p* ^a^	Total (*n* = 7704)	Transition out on health grounds (*n* = 376)
Age at baseline (M, SD)	52.9 (7.0)	59.5 (8.6)	<0.001	58.3 (8.7)	60.3 (8.7)
Male (%)	75.1	68.3	<0.001	70.0	55.1
Socio-economic status (%)					
High	30.9	32.2		32.0	20.7
Medium	51.1	49.7		50.0	47.9
Low	18.1	18.1	0.52	18.1	32.5
Chronic disease (%)	10.8	17.2	<0.001	16.0	37.8
Smoking history (%)					
Never smoker	46.9	46.9		46.9	41.8
Ex-smoker	31.7	34.6		34.1	30.6
Current/intermittent	21.4	18.5	0.20	19.1	27.7
Heavy alcohol use (%)					
Never	75.4	76.4		75.4	79.5
One observation	9.9	10.6		10.6	6.9
Two/three observations	14.8	17.0	0.003	17.0	13.6
Problem drinker^c^ (%)	11.7	9.8	0.04	10.1	14.9
Low physical activity (%)					
Never	16.2	19.1		18.6	33.5
One observation	27.9	29.6		29.3	30.1
Two/three observations	55.8	51.3	0.001	52.1	36.4
Poor diet (%)					
Never	56.5	63.1		61.9	58.0
One observation	17.3	16.6		16.7	16.2
Two/three observations	26.2	20.3	<0.001	21.4	25.8
Physical health status^b^ (M, SD)	51.6 (7.0)	48.6 (9.5)	<0.001	49.1 (0.2)	40.0 (12.4)
Mental health status^b^ (M, SD)	51.5 (9.0)	52.2 (9.2)	0.02	52.1 (9.2)	47.2 (12.1)

*Note*

^a^p value for linear trend

^b^Mean value across follow-up

^c^Problem drinking data available in 1991/93 for a subsample of 7747 participants

Table 2 illustrates the association between accumulated unhealthy behaviours patterns in midlife and transitions out of work, first minimally adjusted for age and then additionally adjusted for the three other health behaviours, socio-economic status and chronic disease at baseline. After adjustment for covariates, male current/intermittent cigarette smokers at midlife were more likely to transition out of work (OR = 1.42, 95% CI 1.18, 1.70). Persistent heavy alcohol drinkers were also more likely to leave work (OR = 1.23, 95% CI 1.03, 1.45) compared to moderate drinkers. In a separate model, problem drinking at baseline was also associated with leaving work over follow-up (OR = 1.27, 95% CI 1.04, 1.54).

**Table 2:** Accumulated exposure to unhealthy behaviours in midlife and subsequent transition out of work

	Men (*n* = 5392)				Women (*n* = 2312)			
	Transition out of work (*n* = 4324)		Transition out on health grounds (*n* = 207)		Transition out of work (*n* = 1958)		Transition out on health grounds^a^ (*n* = 169)	
	Minimally adjusted	Additionally adjusted	Minimally adjusted	Additionally adjusted	Minimally adjusted	Additionally adjusted	Minimally adjusted	Additionally adjusted
Smoking history								
Ex-smoker	1.06 (0.92, 1.21)	1.02 (0.89, 1.17)	1.21 (0.56, 2.60)	1.09 (0.45, 2.62)	1.07 (0.85, 1.34)	1.06 (0.84, 1.32)	1.04 (0.83, 1.30)	1.02 (0.81, 1.28)
Current/intermittent smoker	**1.49 (1.24, 1.78)**	**1.42 (1.18, 1.70)**	**3.67 (1.59, 8.51)**	**3.23 (1.22, 8.55)**	0.81 (0.63, 1.03)	0.82 (0.64, 1.05)	0.79 (0.62, 101)	0.79 (0.62, 1.02)
Heavy alcohol use								
One observation	0.99 (0.81, 1.21)	0.96 (0.78, 1.18)	1.23 (0.41, 3.71)	1.21 (0.33, 4.36)	1.16 (0.81, 1.65)	1.13 (0.79, 1.61)		
Two/three observations	**1.23 (1.03, 1.46)**	**1.23 (1.03, 1.45)**	1.95 (0.81, 4.73)	2.66 (0.96, 7.40)	0.81 (0.58, 1.14)	0.80 (0.57, 1.13)		
No alcohol consumption								
One observation	**1.25 (1.02, 1.54)**	1.20 (0.98, 1.47)	**3.75 (1.42, 9.89)**	**4.97 (1.59, 15.5)**	1.11 (0.84, 1.45)	1.10 (0.84, 1.43)		
Two/three observations	**1.25 (1.02, 1.53)**	1.19 (0.97, 1.46)	**9.65 (3.88,24.02)**	**10.68 (3.70, 30.82)**	1.05 (0.83, 1.33)	1.11 (0.87, 1.41)		
Low physical activity								
One observation	1.03 (0.84, 1.25)	1.00 (0.82, 1.21)	2.88 (1.15, 7.19)	1.95 (0.65, 5.81)	1.02 (0.81, 1.30)	1.07 (0.84, 1.35)	1.02 (0.80, 1.30)	1.07 (0.84, 1.36)
Two/three observations	1.11 (0.88, 1.39)	1.11 (0.88, 1.41)	**6.45 (2.44, 17.0)**	**3.44 (1.07, 11.07)**	1.07 (0.84, 1.36)	1.13 (0.89, 1.43)	1.07 (0.84, 1.35)	1.14 (0.90, 1.45)
Poor diet								
One observation	0.99 (0.83, 1.17)	0.96 (0.81, 1.13)	0.78 (0.31, 1.95)	0.60 (0.20, 1.75)	0.87 (0.67, 1.13)	0.90 (0.69, 1.16)	0.87 (0.67, 1.13)	0.88 (0.68, 1.15)
Two/three observations	0.93 (0.80, 1.09)	0.90 (0.77, 1.05)	1.05 (0.49, 2.24)	1.02 (0.42, 2.49)	**1.31 (1.00, 1.72)**	**1.31 (1.01, 1.72)**	**1.34 (1.02, 1.75)**	**1.33 (1.01, 1.74)**
Problem alcohol drinking (1991/93 only)								
Yes	**1.28 (1.05, 1.35)**	**1.27 (1.04, 1.54)**	**2.87 (1.23, 6.70)**	**3.21 (1.25, 8.21)**	0.93 (0.64, 1.35)	0.90 (0.62, 1.31)	0.93 (0.64, 1.35)	0.90 (0.62, 1.31)

*Note*. Minimally adjusted for age, additionally adjusted for other health behaviours, socio-economic status and prevalent chronic disease. Reference group = no observations

^a^For women, results refer to model containing problem alcohol drinking (insufficient numbers of heavy alcohol drinkers)

Odds ratios that are statistically significant at *p* < 0.05 are highlighted in bold

These associations in men were stronger when considering transitions out of work specifically on health grounds, for smoking (OR = 3.23, 95% CI 1.22, 8.55), heavy alcohol consumption (OR = 2.66, 95% CI 0.96, 7.40) and problem alcohol drinking (OR = 3.21, 95% CI 1.25, 8.21). However, men not drinking alcohol at one observation (OR = 4.97, 95% CI 1.59, 15.5) or at two/three observations (OR = 10.68, 95% CI 3.70, 30.80) were also more likely to transition out of work on health grounds, as were those with consistently low levels of physical activity (OR = 3.44, 95% CI 1.07, 11.07). Confidence intervals are wider for these estimates because the number of men transitioning out of work on health grounds is relatively small.

In women, a consistently poor diet was associated with transitioning out of employment (OR = 1.31, 95% CI 1.01, 1.72). There was no association between other health behaviours and work exit. It was not possible to estimate the association between heavy alcohol use and work exit on health grounds for women, due to small numbers of heavy drinking women. In a model containing problem drinking however, there was no association between problem drinking and work exit on health grounds (Table 2). To evaluate the practical significance of the effect sizes observed, we compared the coefficient for consistently unhealthy behaviours to the coefficient for age. In men, the effect size for smoking, heavy alcohol use and problem drinking were equivalent to 1.2, 0.7 and 0.8 years of advancing age. In women, the effect size for poor diet was equivalent to 0.9 years of advancing age. These effects were similar when restricted to those age under 60.

In sensitivity analyses, the pattern of results was unaffected after excluding observations referring to a second or third transition out of employment, and was similar for transitions out of full time work compared to full or part time. Associations were stronger after excluding participants who changed their health behaviours, suggesting that continued exposure to unhealthy behaviours further increased the association with work exit. Results were not materially different when the models were repeated only participants with complete data, suggesting that missing data or loss to follow-up did not distort the results.

The extent to which physical and mental health status might explain or account for the associations between unhealthy behaviours and transitions out of employment is shown in Additional file 1. In men, physical health status over follow-up explained 19.4% of the contribution of smoking, 15.8% of that for heavy alcohol use and 22.7% of the association for problem alcohol drinking. In women, physical health status explained 6.9% of the association between poor diet and work exit. The role of both physical and mental health status was stronger for work exit on health grounds (range 70.1 to 97.0% of associations explained in men, 79.6% explained in women). There was very little change in the pattern of results in supplementary models (nots shown) considering possible antecedent variables or covariates: childhood adversity (range 2.0 to 11.0% attenuation for health behaviours), cognitive ability (3.1 to 10.0% attenuation), or ethnic minority status (2.1 to 4.8% attenuation). There was no evidence that associations differed for ethnic minority groups.

## Discussion

In a cohort of more than 7700 adults followed from midlife to old age, we identified unhealthy behaviours in midlife associated with subsequent transitions out of employment. Men who smoked cigarettes and consistently drank alcohol heavily (vs. moderately) at midlife, were more likely to leave employment, as were men with problem alcohol drinking. These patterns were stronger when we considered work exit specifically on health grounds. Not drinking alcohol (vs. moderate consumption) was also associated with work exit on health grounds, although this heterogeneous group is known to include participants with significantly higher levels of chronic disease and ‘sick quitters’ [32]. The association between not drinking alcohol and work exit on health grounds was nearly fully accounted for by health-related functioning over follow-up. In women, a consistently poor diet was associated with leaving employment. Weaker associations were observed when the unhealthy behaviours were more occasional, and stronger associations were observed among participants who continued to engage in unhealthy behaviours over follow-up, suggesting that behaviour change (even movement from consistent to occasional use) is a potentially modifiable risk factor for work exit. The size of the contribution from each behaviour was broadly equivalent to one additional year of employment.

Strengths of the study include the large sample size, well-characterised cohort, and length of follow-up. All participants belonged to the same pension scheme, reducing the influence of variation across different pension schemes that might have influenced decisions to retire, although some departments were restructured or privatised which may have influenced these decisions [33]. Repeated measures of health behaviours at midlife allowed us to consider how accumulated exposure to unhealthy behaviours might influence future employment patterns. Several sensitivity analyses suggested that results were robust. One limitation of the study is that results do not generalise to manual occupations or the unemployed at midlife, or those not in work for family reasons. Effects may have been underestimated for smoking and slightly overestimated for alcohol use and diet, due to missing data and attrition. Repeated measures of problem alcohol drinking were not available prior to our study baseline, which may have led us to underestimate the impact of problem drinking on subsequent work exit. Health behaviours were self-reported, which also have led us to underestimate the true impact compared to objective assessments which would reduce measurement error and recall bias. It is not clear why smoking was associated with work exit in men but not women. One explanation is the fact that men in this cohort are known to smoke more and drink more alcohol than the women [34], meaning that women accumulated less unhealthy behaviour, another is the smaller sample size available. Among women, there were fewer with high socio-economic status and these tended to drink alcohol more heavily [26]. We were not able to evaluate drug use, which might also influence work exit and co-occur with unhealthy behaviours. We did not consider income and wealth, but did adjust for civil service employment grade which is a good overall indictor of socio-economic status including income. In the UK, healthcare is universally available without charge which mitigiates concerns to some extent, that results may be driven by residual confounding by income. We considered childhood circumstances and cognitive ability as possible antecedent determinants of the associations seen, but found little attenuation of the effect sizes after aditional adjustment.

Future studies will need to consider additional mechanisms which connect unhealthy behaviours to work exit. Although health-related functioning is one obvious mechanism, particularly for work exit on health grounds, physical and mental health functioning only partly account for work exit in general. Other possible mechanisms that should be considered in future research are the role of unhealthy behaviours in cognitive function, performance at work, satisfaction at work, exposure to work related stressors, personal finances, social networks and time management. A variety of factors occurring over time may influence an individual’s decision to retire. Additionally, retirement age in the wider population is determined by a complex set of factors including government policy, organisational characteristics, and individual choice [35, 36].

## Conclusions

There are several practical policy recommendations that could be made based on the pattern of results seen here. Governments and employers have a role to play in promoting health behaviours among adults of working age [37]. This may involve workplace interventions that discourage sedentary behaviour, promote physical activity, and provide support for individuals who need to reduce their alcohol consumption or modify their drinking behaviour [38]. Health behaviours appear to influence ‘working life expectancy’ [3] and should therefore be of considerable interest to employers and the labour market more generally. Economic modelling has shown that state interventions (e.g. changing eligibility for state pensions, increases to state retirement age [3, 39]) may not be sufficient if people withdraw from the labour market early on health grounds [40]. Public health messages may need to emphasise short and long term consequences of unhealthy behaviour, not just on health but on employment, income and retirement options.

## Additional file

Additional file 1: Table S1. Description of data: Percentage reduction in the association between unhealthy behaviours and transition out of employment after adjustment for physical and mental health functioning over follow-up. (DOCX 12 kb)

## Abbreviations

CAGE: Cutting down, annoyance by criticism, guilty feeling, and ‘eye-opener’ (CAGE) questionnaire
MCS: Mental Component Summary of the SF-36
PCS: Physical Component Summary of the SF-36
SF-36: SF-36 Short Form Health Survey-36
