# Supplementary material for: Association between midlife health behaviours and transitions out of employment from midlife to early old age: Whitehall II cohort study
Source: BMC Public Health. 2017 Jan 17;17:82. doi: 10.1186/s12889-016-3970-4 (PMC5240357; doi:10.1186/s12889-016-3970-4)
Supplement: Additional file 1: Table S1. — Description of data: Percentage reduction in the association between unhealthy behaviours and transition out of employment after adjustment for physical and mental health functioning over follow-up. (DOCX 12 kb) [file 12889_2016_3970_MOESM1_ESM.docx]

Additional file 1: Table S1. Percentage reduction in the association between unhealthy behaviours and transition out of employment after adjustment for physical and mental health functioning over follow-up.

|  | Men | | | | | | Women | | | | | |
| --- | --- | --- | --- | --- | --- | --- | --- | --- | --- | --- | --- | --- |
|  | Transition out of work | | | On health grounds | | | Transition out of work | | | On health grounds | | |
|  | Logit | PCS | MCS | Logit | PCS | MCS | Logit | PCS | MCS | Logit | PCS | MCS |
| Current/intermittent smoker | 0.36 | 0.29 (19.4%) | 0.37 | 0.97 | 0.29 (70.1%) | 0.37 (61.9%) |  |  |  |  |  |  |
| No alcohol use^a^ |  |  |  | 1.63 | 0.12 (92.6%) | 0.15 (90.8%) |  |  |  |  |  |  |
| Heavy alcohol use^a^ | 0.19 | 0.16 (15.8%) | 0.20 |  |  |  |  |  |  |  |  |  |
| Low physical activity^a^ |  |  |  | 1.33 | 0.04 (97.0%) | 0.13 (90.2%) |  |  |  |  |  |  |
| Poor diet^a^ |  |  |  |  |  |  | 0.29 | 0.27 (6.9%) | 0.30 | 1.47 | 0.27 (81.6%) | 0.30 (79.6%) |
| Problem alcohol drinking | 0.22 | 0.17 (22.7%) | 0.26 | 0.70 | 0.17 (75.7%) | 0.26 (62.9%) |  |  |  |  |  |  |

*Note*. Percentage reduction (in brackets) refers to reduction in the log odds (logit) from a subsample of participants with complete data on physical and mental health functioning over follow-up.
